# Amniotic extracellular vesicles’ effect on equine endometrial cells challenged with LPS: a proteomic analysis

**DOI:** 10.20517/evcna.2025.172

**Published:** 2026-05-19

**Authors:** Giulia Gaspari, Alessio Soggiu, Paola Gagni, Salvatore Desantis, Fausto Cremonesi, Anna Lange-Consiglio

**Affiliations:** ^1^Department of Veterinary Medicine and Animal Science (DIVAS), Laboratory of Reproduction and Regenerative Medicine, Università degli Studi di Milano, Lodi 26900, Italy.; ^2^Dipartimento di Scienze Biomediche, Chirurgiche e Odontoiatriche, Università degli Studi di Milano, Milan 20133, Italy.; ^3^Istituto di Scienze e Tecnologie Chimiche “Giulio Natta” (SCITEC), Consiglio Nazionale delle Ricerche (CNR), Milan 20133, Italy.; ^4^Department of Regenerative and Precision Medicine-Jonian Area (DiMePRe-J), University of Bari Aldo Moro, Bari 70124, Italy.; ^#^These authors contributed equally to this work.

**Keywords:** Equine endometrium, inflammation, amniotic extracellular vesicles, proteomics

## Abstract

**Aim:** A balanced uterine environment is crucial for the positive outcome of a pregnancy. Any alteration of its physiological status could affect embryo-maternal crosstalk and impair the mare fertility, as occurs in endometrial inflammatory conditions. The use of regenerative medicine strategies, such as extracellular vesicles (EVs), is emerging as promising therapeutic alternatives. This study aimed to identify the proteins involved in the anti-inflammatory action of equine amniotic EVs on endometrial cells (ECs) challenged by lipopolysaccharide (LPS).

**Methods:** An *in vitro* model of endometrial inflammation was set up by stressing ECs with 10 ng/mL LPS for 3 h. Subsequently, LPS-treated cells were incubated with 400 × 10^6^ EVs/mL for 24 h.

**Results:** The nano-liquid chromatography–high-resolution mass spectrometry (nLC-HRMS) approach identified 794 differentially regulated proteins with *P* < 0.05 and |Log2FC| > 1. Treatment with amniotic EVs entailed an overexpression of several proteins involved in the inflammatory response, including annexins, extracellular matrix (ECM) proteins, antioxidant enzymes and mediators, and transcription factors and regulators. Conversely, other pro-inflammatory mediators were also underexpressed, including cytokines, oxidative factors and matrix metalloproteinase inhibitors.

**Conclusion:** These data suggest that amniotic EVs may exert an anti-inflammatory action on LPS-stressed ECs by multiple mechanisms, including cytokine regulation, antioxidant protection, transcriptional regulation, ECM remodeling and alteration of other key signaling mediators involved in the inflammatory response. These findings could provide useful information on the proteins involved in EVs effect during the initial 24 h of their incorporation into ECs.

## INTRODUCTION

The uterine environment is functionally adapted to support and sustain pregnancy. This communication begins with the reception of sperm, followed by the establishment of immunological tolerance toward the embryo, its implantation, and subsequent support of foetal development^[1]^. These processes require a fine balancing of the microenvironment to guarantee a correct dialogue between maternal and foetal counterparts. A complex sequence of signalling events, comprising a wide range of molecular mediators, is required to ensure this embryo-maternal cross-talk, which is vital for pregnancy establishment and progression^[2,3]^.

Therefore, any transient or pathological alteration of this complex equilibrium may impair pregnancy success^[4]^. This is the case for inflammatory conditions such as endometritis, considered a significant cause of reduced fertility in mares^[5]^. Insemination procedures - both natural and artificial - can trigger an acute inflammatory endometrial reaction, which is generally resolved within a few days. However, repeated inflammatory insults, together with individual susceptibility and increased age, are associated with the development of chronic and more severe forms of endometritis. These conditions often result in endometrial fibrosis and embryo loss^[5]^.

Common therapeutic approaches - including uterine lavages, ecbolic agents, anti-inflammatory or antibiotic drugs - are not always effective in preventing or resolving these conditions, particularly when structural damage, such as fibrosis and endometrial dysfunction, has already occurred^[6]^. Nevertheless, uterine receptivity may be improved or restored through regenerative medicine strategies. While these approaches traditionally relied on the transplantation of mesenchymal stromal/stem cells (MSCs), increasing attention is now being directed towards cell-free solutions. The limited engraftment efficiency of MSCs, together with evidence that their regenerative properties are largely mediated by their secreted factors, have shifted the spotlight toward MSC conditioned medium (CM) and extracellular vesicles (EVs), which constitute the cellular secretome^[7-9]^.

The insoluble fraction of CM is represented by EVs. They are lipidic bi-layered nanosized particles (30-1,000 nm) secreted by cells as key mediators of paracrine signaling. They carry a varied molecular cargo composed of lipids, proteins and nucleic acids^[10]^. Upon delivery and internalization by recipient cells, EVs can alter cell biological function^[11]^, thus playing a critical role in cell-to-cell communication and tissue regeneration.

Extra-fetal adnexa are emerging as a valuable source of MSCs due to their ethical and non-invasive collection, differentiation potential and scarce immunogenicity. Adnexal MSCs preserve characteristics of the primitive layers and are considered intermediate between embryonic and adult stem cells^[12]^. Evidence indicates that amniotic-derived CM can improve endometrial cell proliferation^[13]^, while amniotic EVs can down-regulate tumor necrosis factor-a (TNF-α), interleukin 6 (IL-6), matrix metalloproteinase-1 (MMP-1) and MMP-13, while restoring transforming growth factor-b (TGF-b) expression in equine endometrial cells (ECs)^[14]^. These findings suggest a potential role in endometrial regeneration. Moreover, their surface glycopattern facilitates their efficient internalization by equine ECs^[15]^, enabling the transfer of EV molecular cargo to target ECs. The microRNA (miRNA) content of these vesicles has already been characterized, reveling a selective compartmentalization of specific miRNAs involved in inflammatory and immune response modulation^[16]^. Amniotic EVs can also be administered *in vivo* to achieve an immunomodulatory action. Indeed, their intrauterine infusion proved effective in preventing the onset of persistent post-breeding endometritis^[17]^.

Proteomic studies concerning mare endometritis have primarily aimed to elucidate the pathological mechanisms leading to reproductive disorders. Most investigations have focused mainly on uterine fluid, analyzing the proteome of healthy cycling^[18]^, pregnant^[19]^ or endometritis-affected mares^[20,21]^.

In parallel, increasing number of studies are focusing on the proteomic content of regenerative medicine products, in order to deepen their therapeutic capabilities. Mesenchymal stromal cells, CM, or EVs - whether analyzed individually or comparatively - have been demonstrated to exhibit distinct proteomic signatures^[22-24]^, albeit still expressing markers reflecting their common origins^[24]^. De Moraes *et al*. investigated the secretome of bovine endometrial mesenchymal progenitor/stem cells following lipopolysaccharide (LPS) stimulation^[25]^. Their results revealed positive enrichment for proteins involved in antibacterial response, macrophage activation, receptor-mediated endocytosis, hydrolase activity and inhibitory enzymes. Additionally, proteins with antimicrobial, anti-inflammatory and tissue remodeling properties were identified both in LPS-challenged and control secretomes, highlighting the therapeutic potential of these cell-derived products.

However, to the best of the authors’ knowledge, studies investigating the therapeutic effect of EVs on the proteome of inflamed cells - particularly within the equine endometrium - are still missing. Consequently, the specific mechanisms through which amniotic EVs exert their immunomodulatory and regenerative effects remain to be fully elucidated. Therefore, the aim of the present *in vitro* study was to investigate the proteins involved in the action of amniotic EVs, by evaluating the effects of their incorporation into equine ECs under LPS-induced inflammation using a proteomic approach.

## METHODS

### Reagents

All reagents were obtained from Sigma-Aldrich (Milan, Italy), whereas test tubes and culture plates were supplied by Euroclone (Milan, Italy).

### Endometrial cell isolation and culture

Equine uteri (*n* = 3) were collected at the abattoir and transported to the laboratory on ice. The animals were sacrificed for reasons unrelated to this study and were intended for human consumption. The uteri were selected from post-pubertal mares in the dioestrus phase, based on the presence of an obvious corpus luteum and absence of detectable genital disease.

Uteri samples were processed according to Perrini *et al*.^[14]^. The horn ipsilateral to the corpus luteum was opened to allow endometrial washing with sterile saline. Endometrial strips were cut longitudinally using scissors, avoiding the underlying connective tissue. Endometrial samples were then digested for 3 h at 38.5 °C in sterile Hank’s buffered salt solution (HBSS) supplemented with 1 mg/mL collagenase II, 0.4 mg/mL DNase I and 4 mg/mL bovine serum albumin (BSA). The product of digestion was passed through a filter (80 μm pores), centrifuged at 200 × *g* for 10 min and washed twice in phosphate buffer solution (PBS).

The obtained cells were seeded at a density of 1 × 10^5^ cells/cm^2^ in high glucose Dulbecco's Modified Eagle's Medium (HG-DMEM) supplemented with 10% fetal bovine serum (FBS), amphotericin B (0.25 μg/mL), penicillin (100 UI/mL), streptomycin (100 μg/mL), and 2 mM L-glutamine. Cultures were maintained in humidified incubator at 38.5 °C with 5% CO_2_. After 18 h of incubation, cellular medium containing the epithelial population of ECs was removed and reseeded, while the connective population remained adherent to the culture flask. Once they reached sub-confluence, both cell populations were detached with 0.05% trypsin-Ethylenediaminetetraacetic acid (EDTA) and cryopreserved at passage (P) 1. For this study, only endometrial epithelial cells (ECs) at P1 were used.

### Equine amniotic mesenchymal stromal cell isolation and culture

After spontaneous and physiological deliveries, three allanto-amniotic membranes, normally discarded at parturition, were collected from healthy mares bred for purposes external to this study. All procedures were performed in accordance with standard veterinary practice and in compliance with 2010/63 European (EU) directive on animal protection.

Allanto-amniotic membranes were transported to the laboratory in sterile physiological saline at +4 °C and processed within 12 h from the collection. The amnion was mechanically detached from the underlying allantois, sectioned longitudinally, and incubated for 9 min at 38 °C in PBS containing 2.4 U/mL dispase (Becton Dickinson & Company, Milan, Italy). Amniotic fragments were initially incubated for 5-10 min at room temperature (RT) in HG-DMEM supplemented with 10% FBS and 2 mM L-glutamine (Euroclone, Milan, Italy). This step was followed by enzymatic digestion for 3 h using 1 mg/mL collagenase type I and 20 mg/mL DNase (Roche, Mannheim, Germany) at 38.5 °C. The resulting digested material was passed through a 100 µm pore-size mesh filter and amniotic mesenchymal stromal cells (AMCs) were harvested by centrifugation at 250 × *g* for 10 min. For the first passage, AMCs were seeded at a density of 1 × 10^5^ cell/cm^2^, while subsequent passages were performed at a density of 1 × 10^4^ cell/cm^2^ in complete medium supplemented with 10 ng/mL epidermal growth factor and incubated in humidifier incubator at 38.5 °C with 5% of CO_2_.

The isolation of these cells is a standardized procedure in this laboratory. In addition to their characterization in terms of differentiation potential, mesenchymal markers, and absence of immunogenicity, these cells have also been investigated for their miRNA cargo^[16]^.

### CM production

Upon reaching P 3, AMC culture medium was replaced with serum-free ultra-culture medium (Ultraculture, Lonza, Milan, Italy) and maintained under these conditions for 72 h. The CM was collected every 24 h and replaced with fresh serum-free medium. Cell contamination was removed by centrifugating CM at 1,600 × *g* for 20 min, while any remaining debris was discarded by a further 4,500 × *g* centrifugation for 20 min. Obtained CM was then stored at -80 °C until EV isolation.

### EV isolation

EVs were isolated by ultracentrifugation of CM at 100,000 × *g* for 1 h at 4 °C using an OptimaX system (Beckman Coulter, Milan, Italy). The resulting pellet was resuspended in serum-free medium and, following determination of EV concentration and size distribution by NanoSight instrument, aliquoted and stored at -20 °C until further use. Similar to AMCs, EV isolation procedures are well standardized in this laboratory. Vesicle characterization is routinely performed in accordance with Minimal Information for Studies of Extracellular Vesicles (MISEV) guidelines^[10]^ as previously described^[17]^, using NanoSight analysis for EV concentration and size, Western blot for the detection of EV-associated markers and transmission electron microscopy (TEM) for morphological assessment.

Previously, equine EV miRNA cargo^[16]^ and equine EV proteomic profile compared to AMCs^[26]^ have already been studied.

### Extracellular vesicle nanoparticle tracking analysis (NTA)

Extracellular vesicle size distribution and concentration were determined by NTA using a NanoSight NS300 instrument (Malvern Panalytical, Malvern, UK) equipped with a 532 nm laser, in accordance with the manufacturer’s instructions. Three samples were introduced via a syringe pump operating at a constant flow rate. For each sample, three 60 s videos were captured and subsequently analyzed using version 3.4 of the NTA software. The mean, mode, and median particle diameters obtained from each recording were used to calculate EV concentration, expressed as nanoparticles/mL.

### Extracellular vesicle bicinchoninic acid assay and Western blot analysis

To ensure the purity of the EV samples, total protein quantification was performed by bicinchoninic acid (BCA) assay accordingly to what previously described in Gaspari *et al*.^[26]^. Then, similarly, Western blot analysis was performed to verify the absence of calnexin, together with the presence of EV-associated protein Alix (internal marker) and the surface markers CD63 and CD81.

Three biological samples containing 20 × 10^9^ EVs were mixed with RadioImmunoprecipitation Assay (RIPA) buffer at a 1:1 ratio and supplemented with Halt^TM^ Protease Inhibitor (1×). Samples were briefly sonicated, gently mixed for 30 min, and subsequently subjected to 14,000 × *g* centrifugation for 15 min. The collected supernatants were transferred to fresh tubes, and Pierce^TM^ BCA Protein Assay was used to determine protein concentration. Prior to measurement, EV samples were diluted 1:5. After correction for dilution factors, the total protein concentration was 431.4 ± 21.7 µg/mL. For Western Blot analysis, EV samples were normalized to the same protein concentration using 5× Laemmli buffer under reducing conditions, allowing the loading of 16 µg of total protein per lane. A 10 µL aliquot of Precision Plus All Blue Protein Standards (Bio-Rad Laboratories S.r.l., Milan, Italy) and 32 µL of each prepared sample were loaded onto 4%-15% Bio-Rad Tris-Glycine eXtended (TGX) gradient gels. Proteins were separated by Sodium Dodecyl Sulfate-Polyacrylamide Gel Electrophoresis (SDS-PAGE) at 220 V and subsequently transferred for Western blot analysis.

To detect EV characteristic markers, Western blotting was performed in triplicate by mixing 8 µL of Laemmli buffer with 32 µL of each sample under reducing conditions, followed by a heating step at 95 °C for 10 min. Following loading onto 4%-20% SDS-PAGE gels (Mini-PROTEAN TGX Precast Protein Gels, Bio-Rad), and separation under an electric field, samples were transferred onto nitrocellulose membranes by a Trans-Blot Turbo system (Bio-Rad, Milan, Italy).

Membranes were blocked at RT for 1 h in TBS-T (150 mM NaCl, 20 mM Tris-HCl, pH 7.4, 0.5% Tween-20) with 5% (w/v) BSA to prevent nonspecific binding. Membranes were then incubated overnight at 4 °C under orbital agitation with the following primary antibodies: monoclonal anti-Alix (1:1,000; Santa Cruz Biotechnology, CA, USA), monoclonal anti-CD81 (1:500; Santa Cruz Biotechnology), and monoclonal anti-CD63 (1:500; BD Biosciences, NJ, USA). A monoclonal anti-calnexin antibody (1:1,000; Sigma) was used as a purity control for EV preparations.

Following three TBS-T washes of 5 min each, membranes were subjected to a 1-h incubation at RT with an anti-mouse secondary antibody conjugated with HRP (Bio-Rad) and diluted 1:3,000 in TBS-T containing 1% BSA. Following additional washes, Clarity^TM^ Western ECL substrate (Bio-Rad) was used to visualize immunoreactive bands, then imaged with a ChemiDoc XRS+ system (Bio-Rad).

### TEM

A 10 µL drop of three samples of EVs (20 × 10^9^ particles/mL each) was adsorbed onto 300-mesh copper grids coated with Formvar/carbon. A 5-min fixation step was performed with 2.5% glutaraldehyde. After several washes with distilled water, 2% uranyl acetate was used to contrast the grids, which were then air-dried, and visualized by a transmission electron microscope. Digital images were acquired.

### Extracellular vesicle incorporation after LPS treatment

Equine ECs at P1 were stressed with LPS in order to mimic an inflammatory status. Equine cells were seeded at 1 × 10^4^ cell/cm^2^ in complete HG-DMEM. Based on the study by Perrini *et al*.^[14]^, cells were treated with 10 ng/mL of LPS (diluted in HG-DMEM) for 3 h at 38.5 °C in a humidified incubator with 5% CO_2_. This concentration was selected as it effectively induced up-regulation of TNF-α, and ILβ-1 expression. Non-stimulated ECs, used as controls and co-incubated with EVs, did not show induction of pro-inflammatory gene expression^[14]^.

After LPS treatment, ECs were washed twice in PBS and subsequently incubated with 400 × 10^6^ EV/mL in HG-DMEM for 24 h at 38.5 °C under standard culture conditions (humidified atmosphere, 5% CO_2_). This concentration was chosen based on the study of Gaspari *et al*.^[15]^, in which it yielded optimal EV internalization as assessed by fluorescence microscopy. EV internalization was confirmed by a rapid fluorescence microscopy assessment. No further investigations were performed, as previous results had already demonstrated consistent cellular response at the selected dose and exposure time.

Three experimental conditions were established: control ECs, LPS-treated ECs (LPS group) and LPS/EV-treated ECs (LPS/EV group).

### Proteomic analysis

#### High resolution mass spectrometry analysis (nLC-HRMS)

Following co-incubation with EVs, ECs were detached by trypsinization and centrifuged at 250 × *g* for 10 min. After two washes in PBS, the obtained cell pellet was centrifuged again to obtain a dry pellet that was stored at -80 °C until protein extraction.

As previously described in Gaspari *et al*.^[26]^, all samples were analyzed by a Dionex Ultimate 3000 nano-LC system (Sunnyvale, CA, USA) connected to Orbitrap Exploris^TM^ 240 Mass Spectrometer (Thermo Scientific, Bremen, Germany) equipped with nano electrospray ion source, at the UNITECH OMICs (University of Milano, Italy). An Acclaim PepMap 100 - 100 μm × 2 cm C18 (Thermo Scientific) was used for pre-concentration of peptide mixtures, which were then separated on EASY-Spray column ES900, 25 cm × 75 μm ID packed with Thermo Scientific Acclaim PepMap RSLC C18, 3 μm, 100 Å. Mobile phase A (0.1 % formic acid in water) and mobile phase B (0.1% formic acid in acetonitrile 20/80, v/v) were used at a flow rate of 0.300 μL/min, at 35 °C. Samples were injected in triplicate. To prevent sample carryover, two blank runs were included between samples. MS spectra were collected over a 375-1,500 Da m/z range, at 120,000 resolution, operating in data-dependent mode, with a 3 sec cycle time between master scans. HCD was performed with collision energy set at 35 eV. Polarity: positive.

#### Bioinformatic analysis

The raw data files were analyzed using MSFragger-DDA+ (version 4.1) (https://www.nature.com/articles/s41467-025-58728-z) coupled with FragPipe (version 22.0) and uniprotkb equus_caballus_reviewed database (20.02.2025). Peptide-spectrum matches were validated using Percolator and protein inference was performed by ProteinProphet, with final protein lists filtered at 1% protein-level False Discovery Rate (FDR) using the picked FDR strategy (--sequential --prot 0.01 --picked). Protein intensity values were extracted from the *combined_protein.tsv* output and imported into RStudio 2025.05.0 (Posit Software, PBC) for independent statistical analysis using a custom R script. Contaminant proteins were filtered out and proteins with < 2 peptides (< 3 valid intensity measurements) were removed. Differential expression analysis was performed using Welch’s two-sample *t*-test for each pairwise comparison (LPS *vs*. ECs, LPS/EV *vs*. ECs, LPS/EV *vs*. LPS). A combined threshold of *P* < 0.05 and |log2 fold change| ≥ 1 was applied to identify differentially expressed proteins, following the dual-threshold approach recommended for exploratory proteomics with limited biological replication^[27]^. Functional protein analysis and visualization were performed using the same R script with libraries *readr*, *dplyr*, *tidyr*, *ggplot2*, *pheatmap*, *RColorBrewer*, *ggrepel*, *patchwork*, *clusterProfiler*, *org.Hs.eg.db*, *ReactomePA*, *stringr*, *gridExtra* and *enrichplot*.

## RESULTS

### Extracellular vesicle characterization

**Figure 1:**
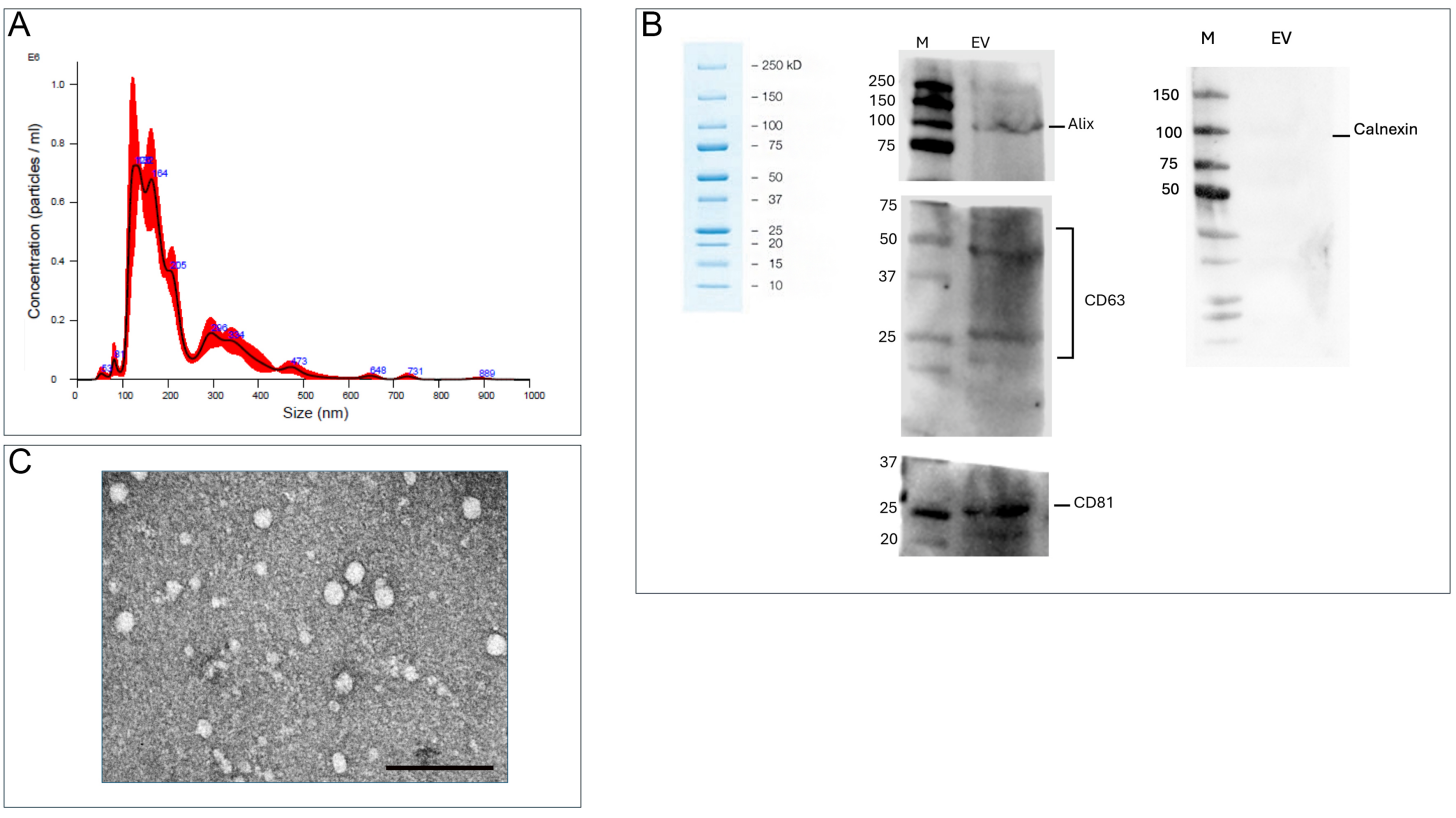
EV characterization. (A) NanoSight analysis of AMC-EV size distribution and concentration; (B) Western blot analysis of the EV-associated protein Alix (internal marker) and the surface markers (CD63 and CD81). Absence of Calnexin indicates lack of cellular contamination. Three biological samples were pooled together to perform Western blot analysis in technical triplicate; (C) TEM of AMC-derived EVs. Scale bar: 1 µm. AMC-EVs: Amniotic mesenchymal cell-derived extracellular vesicles; EVs: extracellular vesicles; TEM: transmission electron microscopy; CD63: cluster of differentiation 63; CD81: cluster of differentiation 81.

The isolated EVs complied with MISEV guidelines^[10]^. NanoSight analysis revealed an average particle size of 216.3 ± 10.0 nm and a concentration of 1.53 × 10^11^ ± 1.79 × 10^9^ particles/mL. Based on these dimensions, the isolated EV population consisted predominantly of microvesicles [Figure 1A].

Western blot analysis confirmed the presence of the EV-associated protein Alix as internal marker, and CD81 and CD63 as membrane-associated markers, thereby validating the extracellular vesicle nature of the preparation [Figure 1B]. In addition, the absence of Calnexin in the EV samples indicates lack of cellular contamination, supporting the efficiency of the EV purification process.

TEM provided further confirmation that the EV isolation protocol was successful, revealing EVs with a characteristic spheroidal morphology [Figure 1C].

### EV characterization and overall proteomic landscape

**Figure 2:**
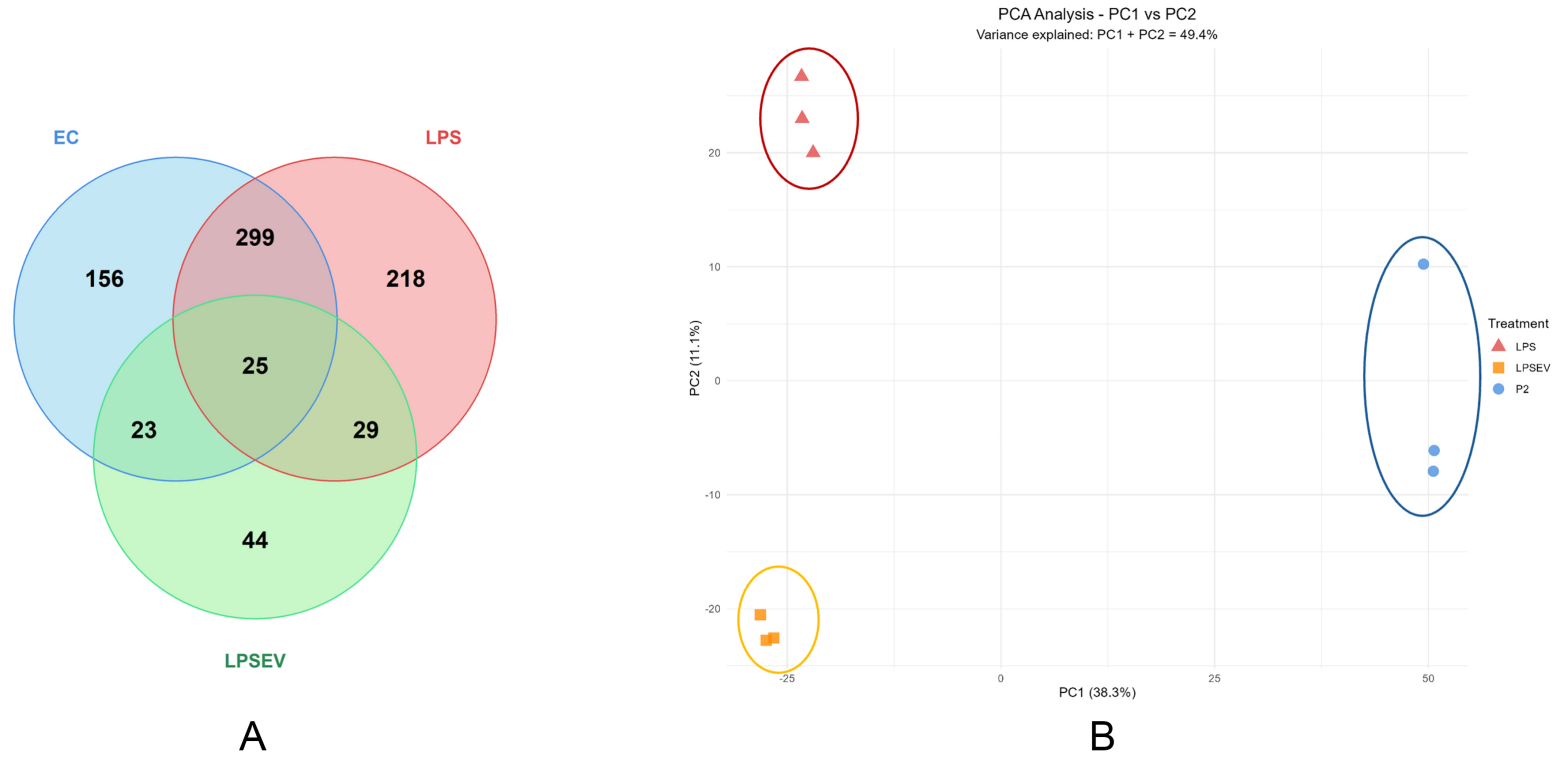
Protein expression characterization of ECs, LPS and LPS/EV groups. (A) Venn diagram illustrating the distribution of significantly regulated proteins (*P* < 0.05, |log2 fold change| > 1) across the three pairwise comparisons: LPS *vs*. ECs (503 proteins), LPS/EV *vs*. ECs (571 proteins), and LPS/EV *vs*. LPS (121 proteins). Numbers indicate proteins unique to each comparison or shared between comparisons; (B) PCA showing different clustering of the three groups. PC1 (46.1% variance) and PC2 (13% variance) separate control ECs (blue ellipse), LPS (red ellipse) and LPS/EV (orange ellipse) groups, indicating substantial proteomic differences among groups. PCA: Principal component analysis; PC1: principal component 1; PC2: principal component 2; ECs: endometrial cells; LPS: lipopolysaccharide; EV: extracellular vesicle; LPS/EV: lipopolysaccharide plus extracellular vesicles combined treatment group.

A total of 3,307 proteins were identified using a cutoff of at least 2 peptides per protein. Differential protein expression analysis revealed distinct and overlapping responses to inflammatory stimuli and extracellular vesicle treatment. A total of 794 proteins showed significant differential expression across all comparisons. The largest overlap occurred between inflammatory responses, with 299 proteins commonly regulated in both LPS *vs*. ECs and LPS/EV *vs*. ECs comparisons, representing the core inflammatory signature shared between direct LPS stimulation and combined LPS+EV treatment. Condition-specific responses were also evident. A total of 156 proteins were uniquely regulated by LPS treatment alone, while 218 proteins showed exclusive regulation in the LPS/EV *vs*. ECs comparison, suggesting distinct molecular pathways activated by the combined treatment. Notably, 44 proteins were specifically modulated by EVs (LPS/EV *vs*. LPS comparison), representing the pure EV-mediated effects independent of the underlying inflammatory stimulus. The central overlap of 25 proteins represents a core set of molecules responsive to all three experimental conditions, potentially identifying key regulatory nodes in the inflammatory-EV interaction network. Smaller overlaps (23 and 29 proteins) between paired comparisons indicate intermediate levels of pathway crosstalk [Figure 2A].

Principal component analysis (PCA) revealed distinct clustering and separation of the three samples [Figure 2B].

**Figure 3:**
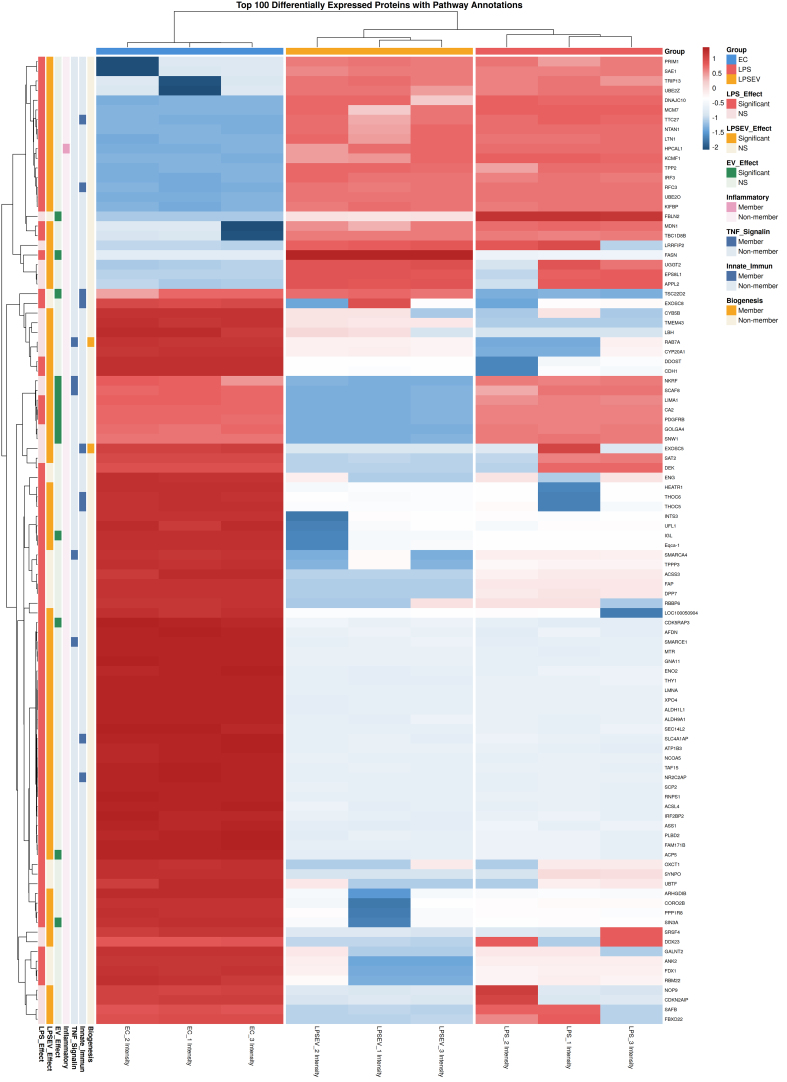
Heatmap visualization of the top 100 differentially expressed proteins among ECs, LPS and LPS/EV groups. Z-score normalized expression values are displayed, with red indicating higher relative expression and blue lower relative expression. Hierarchical clustering was performed on both proteins (rows) and samples (columns). Annotation bars indicate membership in key biological pathways (EV biogenesis, innate immunity, TNF signaling, inflammatory response) and statistical significance of each protein in the three pairwise comparisons. ECs: Endometrial cells; LPS: lipopolysaccharide; EV: extracellular vesicle; TNF: tumor necrosis factor; LPS/EV: lipopolysaccharide plus extracellular vesicles combined treatment group.

**Figure 4:**
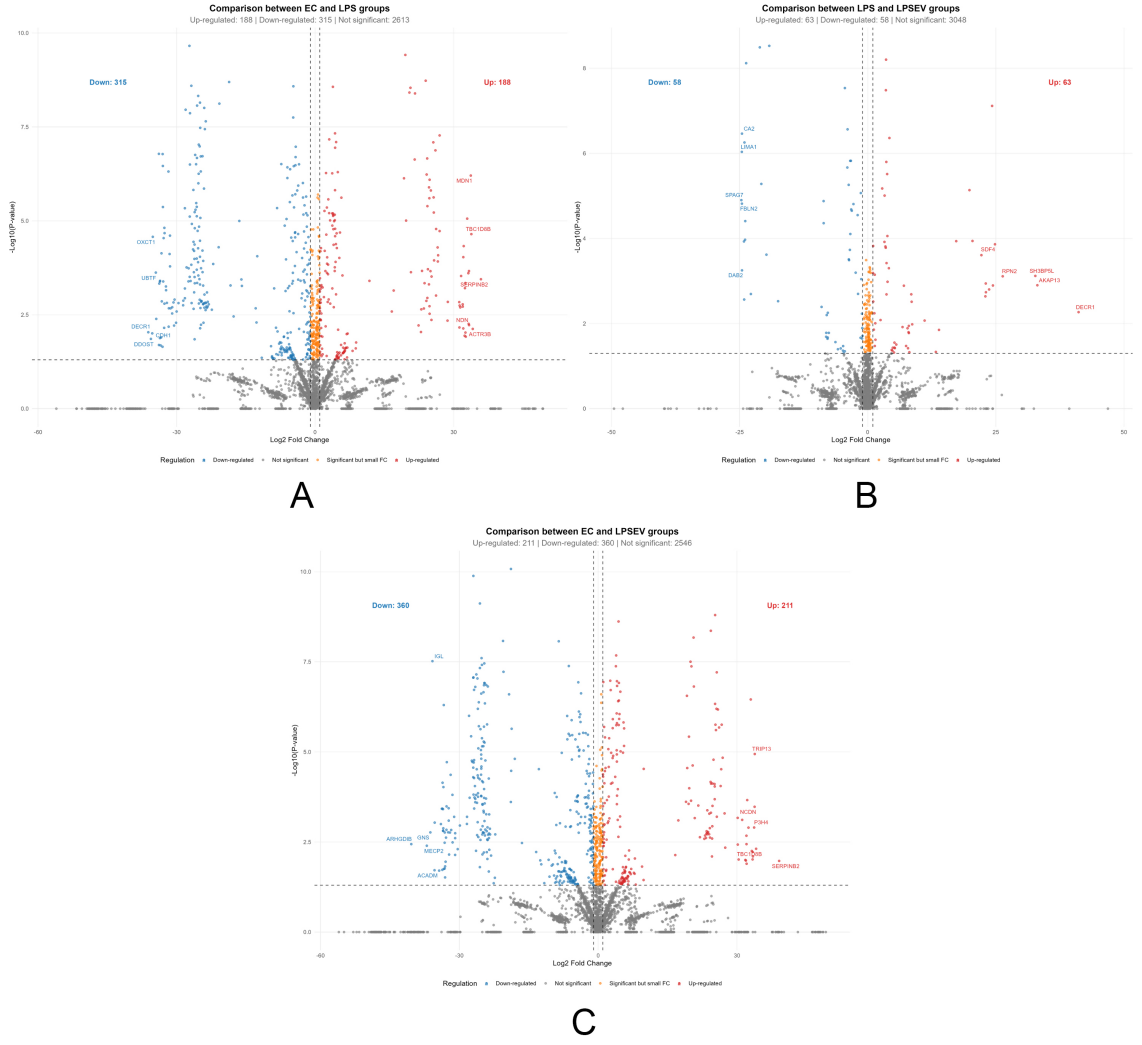
Differentially expressed proteins among groups represented by volcano plots (*P* < 0.05, |log2 fold change| ≥ 1). (A) Comparison between ECs and LPS groups (Up-regulated: 188; Down-regulated: 315); (B) Comparison between LPS and LPS/EV groups (Up-regulated: 63; Down-regulated: 58); (C) Comparison between ECs and LPS/EV groups (Up-regulated: 211; Down-regulated: 360). The x-axis shows log2 fold change, while the y-axis shows -log10 *P* values. Dashed lines indicate significance thresholds. ECs: Endometrial cells; LPS: lipopolysaccharide; EV: extracellular vesicle; LPS/EV: lipopolysaccharide plus extracellular vesicles combined treatment group.

The heatmap showed distinct protein expression profiles, especially for ECs compared to LPS and LPS/EV groups [Figure 3]. Volcano plots display the differentially regulated proteins between ECs and LPS [Figure 4A], LPS and LPS/EV [Figure 4B], ECs and LPS/EV [Figure 4C].

### Gene Ontology enrichment

To investigate the main biological processes triggered by LPS stress and further EV treatment, Gene Ontology (GO) enrichment analysis was performed across all group comparisons.

**Figure 5:**
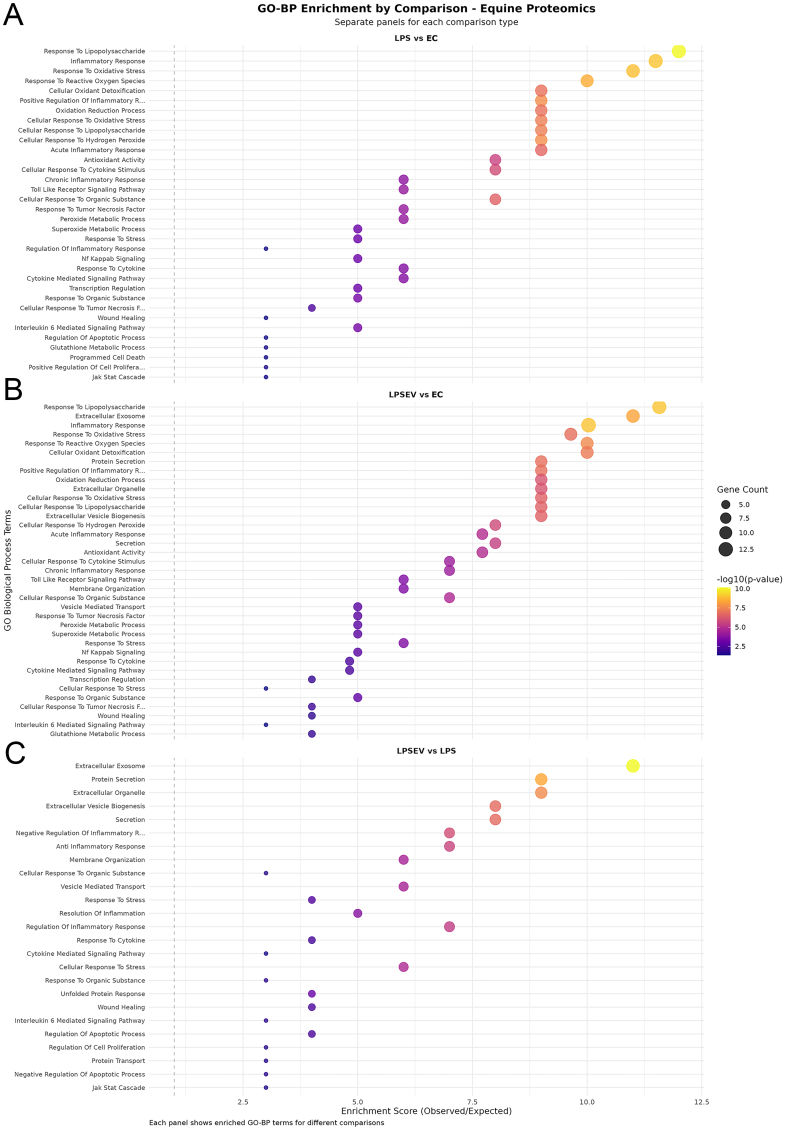
GO-BP enrichment dot plots. (A) Top enriched terms in the LPS *vs*. ECs comparison; (B) Top enriched terms in the LPS/EV *vs*. ECs comparison; (C) Top enriched terms in the LPS/EV *vs*. LPS comparison. Dot size represents gene count; color scale indicates the enrichment *P* value. GO-BP: Gene Ontology Biological Process; ECs: endometrial cells; LPS: lipopolysaccharide; EV: extracellular vesicle; LPS/EV: lipopolysaccharide plus extracellular vesicles combined treatment group.

Compared to the ECs group, the treatment with LPS led to an enrichment in terms involved in inflammation-related mechanisms, such as response to stress, regulation of transcription and signaling of TNF and nuclear factor kappa-light-chain-enhancer of activated B cells (NF-κB) factors, oxidative regulation, and wound healing. Indeed, the most significant terms included “response to LPS”, “inflammatory response”, “response to oxidative stress”, “response to reactive oxygen species”, “cellular oxidant detoxification”, “acute inflammatory response”, “cellular response to cytokine stimulus”, “Toll like receptor signaling pathway”, “response to tumor necrosis factor”, “NFkB signaling” or “IL-6 mediated signaling pathway” [Figure 5A].

The comparison between LPS/EV to ECs group obviously comprised many of the above-mentioned terms, with additional enrichment in pathways related to EVs, namely “extracellular exosome”, “extracellular organelle”, “extracellular vesicle biogenesis”, “membrane organization” and “vesicle-mediated transport” [Figure 5B].

The LPS/EV to LPS group comparison shows the effect of EV incorporation on LPS-stressed cells. As expected, the resulting protein set targeted GO terms related to extracellular vesicle mechanisms; in addition, significant enrichment was observed for terms including “negative regulation of inflammatory response”, “anti-inflammatory response”, “response to stress”, “resolution of inflammation”, “wound healing”, “negative regulation of apoptotic process” and “regulation of cell proliferation” [Figure 5C].

An additional comparison of the most significantly enriched Gene Ontology Biological Process (GO-BP) terms is provided in Supplementary Figure 1.

### Validation of the LPS-induced inflammatory model (LPS *vs.* ECs)

To confirm the effectiveness of the *in vitro* inflammatory model, the proteomic profile of LPS-treated ECs was compared to that of untreated controls. A total of 503 proteins were differentially expressed (*P* < 0.05, |log2 fold change| ≥ 1), of which 188 were upregulated and 315 downregulated by LPS treatment, representing approximately 15.2% of the quantified proteome [Figure 4A]. The magnitude and directionality of these changes, together with the GO enrichment for inflammatory pathways [Figure 5A], validate the model and provide the necessary reference framework against which EV effects are assessed.

**Figure 6:**
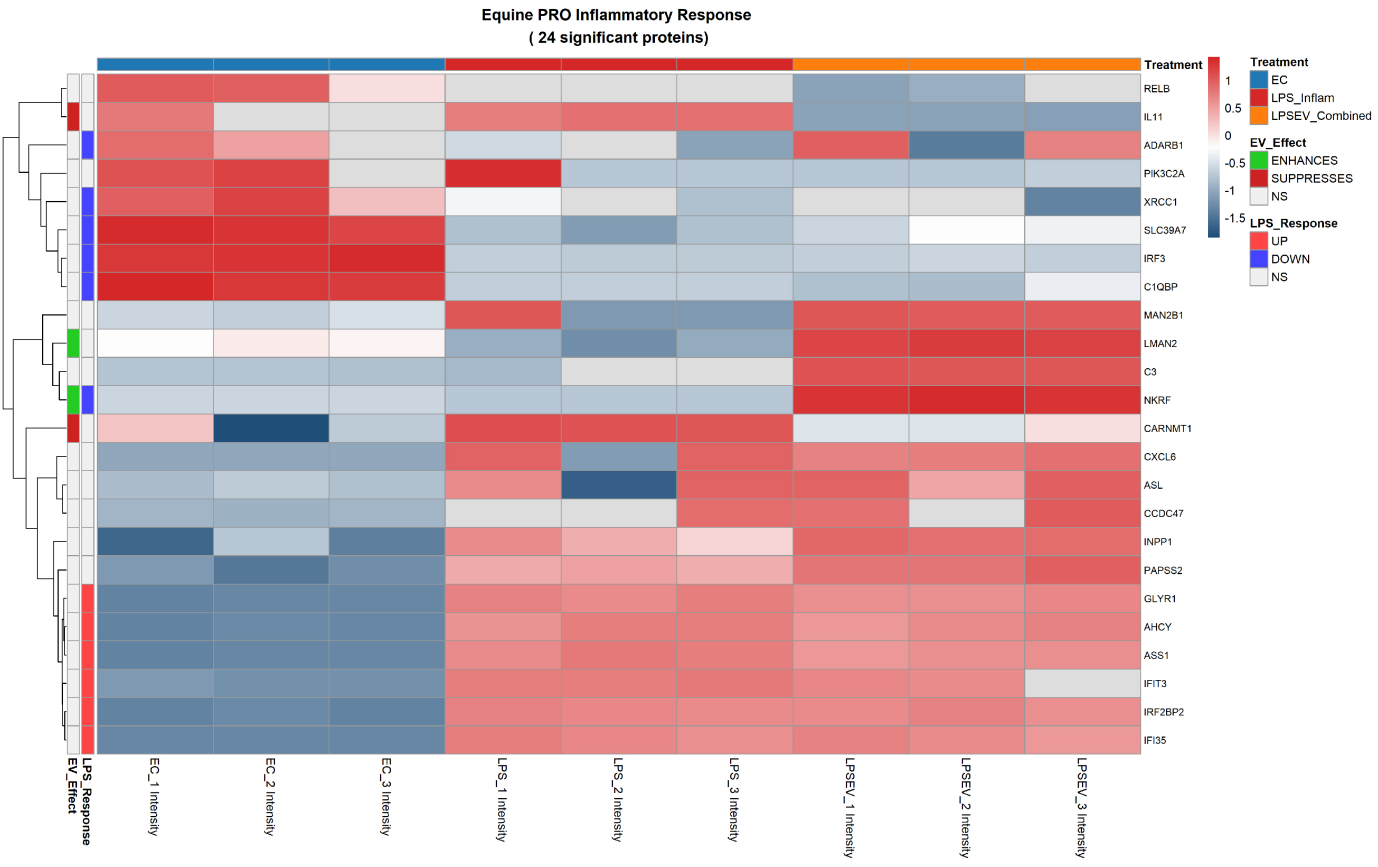
Pro-inflammatory response heatmap of 24 significant proteins differentially expressed among ECs, LPS and LPS/EV groups. Z-score normalized expression values are shown (red: higher, blue: lower). Left annotation columns: EV_Effect (green: enhanced by EVs, red: suppressed by EVs, white: not significant in LPS/EV *vs*. LPS comparison); LPS_Response (red: upregulated by LPS, blue: downregulated by LPS, white: not significant in LPS *vs*. ECs comparison). Upper colored bars indicate experimental groups: light blue (ECs), red (LPS), orange (LPS/EV). ECs: Endometrial cells; LPS: lipopolysaccharide; EVs: extracellular vesicles; NS: not significant; Z-score: standard score; LPS/EV: lipopolysaccharide plus extracellular vesicles combined treatment group.

Challenge with LPS modulated the expression of several proteins involved in inflammatory response and oxidative stress resistance. Some of the most relevant upregulated pro-inflammatory proteins include Glyoxylate Reductase 1 homolog (GLYR1), Adenosylhomocysteinase (AHCY), Argininosuccinate Synthase 1 (ASS1), and Interferon induced and regulatory proteins (IFIT3, IFI35 and IRF2BP2). Conversely, LPS stress down-regulated Interferon Regulatory Factor 3 (IRF3), Complement C1q Binding Protein (C1QBP) and NF-κB Repressing Factor (NKRF), among others [Figure 6].

**Figure 7:**
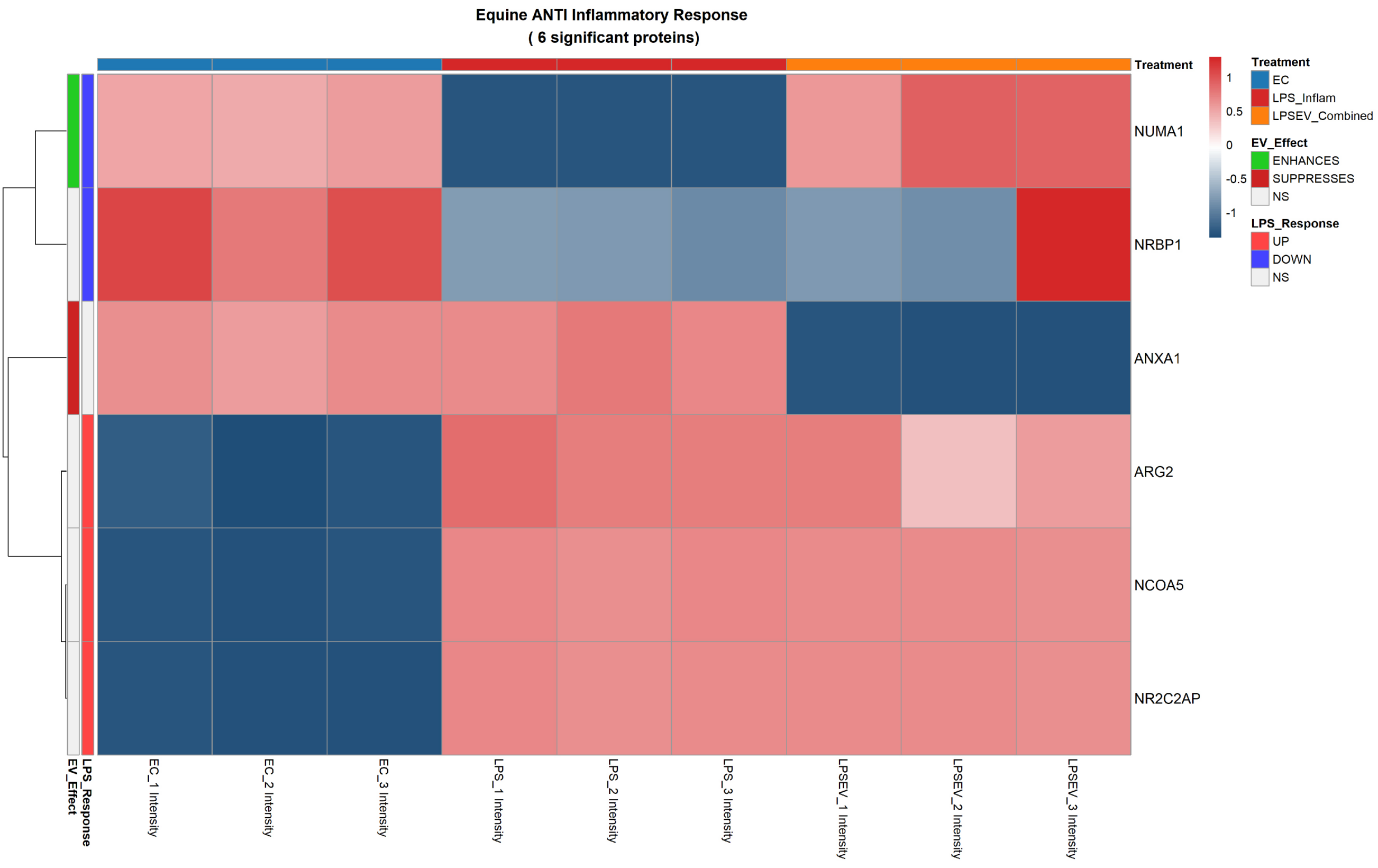
Anti-inflammatory response heatmap of 6 significant proteins differentially expressed among ECs, LPS and LPS/EV groups. Z-score normalized expression values are shown (red: higher, blue: lower). Left annotation columns: EV_Effect (green: enhanced by EVs, red: suppressed by EVs, white: not significant in LPS/EV *vs*. LPS comparison); LPS_Response (red: upregulated by LPS, blue: downregulated by LPS, white: not significant in LPS *vs*. ECs comparison). Upper colored bars indicate experimental groups: light blue (ECs), red (LPS), orange (LPS/EV). ECs: Endometrial cells; LPS: lipopolysaccharide; EVs: extracellular vesicles; LPS/EV: lipopolysaccharide plus extracellular vesicles combined treatment group; NS: not significant; Z-score: standard score.

The regulation of the anti-inflammatory response mediated by LPS stress, included the up-regulation of Arginase 2 (ARG2), Nuclear Receptor Coactivator 5 (NCOA5), and Nuclear Receptor 2C2 Associated Protein (NR2C2AP) and the down-regulation of nuclear mitotic apparatus protein 1 (NUMA1) [Figure 7].

**Figure 8:**
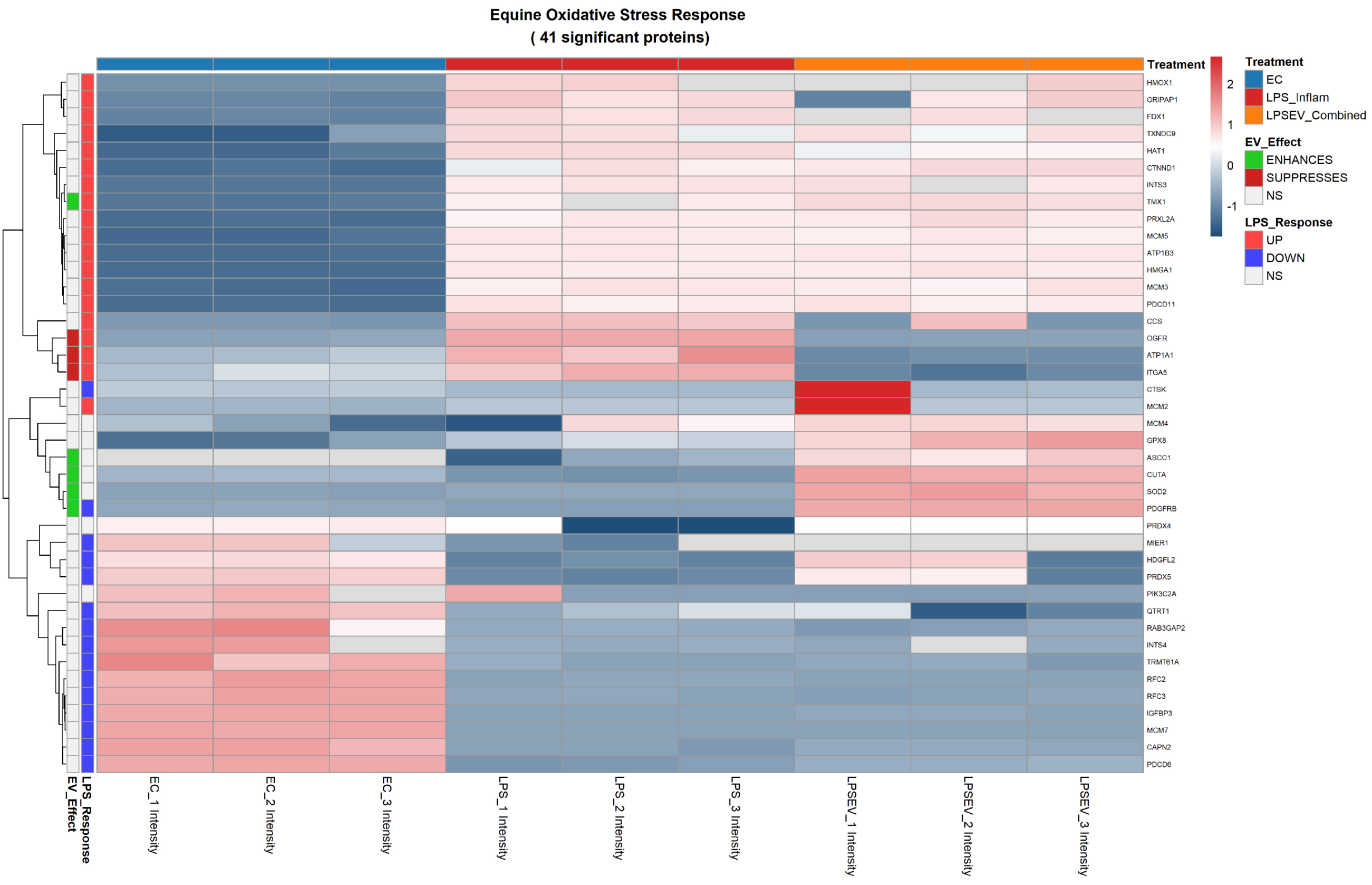
Oxidative stress resistance heatmap of 41 significant proteins differentially expressed among ECs, LPS and LPS/EV groups. Z-score normalized expression values are shown (red: higher, blue: lower). Left annotation columns: EV_Effect (green: enhanced by EVs, red: suppressed by EVs, white: not significant in LPS/EV *vs*. LPS comparison); LPS_Response (red: upregulated by LPS, blue: downregulated by LPS, white: not significant in LPS *vs*. ECs comparison). Upper colored bars indicate experimental groups: light blue (ECs), red (LPS), orange (LPS/EV). ECs: Endometrial cells; LPS: lipopolysaccharide; EVs: extracellular vesicles; LPS/EV: lipopolysaccharide plus extracellular vesicles combined treatment group; EO: equine oxidative stress response; NS: not significant; Z-score: standard score.

Concerning oxidative stress resistance, LPS induced increased abundance of thioredoxin family members [Thioredoxin Domain Containing 9 (TXNDC9), transmembrane protein 1 (TMX1)], DNA damage response proteins of the minichromosome maintenance complex (MCM2, MCM3, MCM5), integrator complex subunit 3 (INTS3), programmed cell death 11 (PDCD11), ATPase Na+/K+ transporting subunit beta 3 (ATP1B3) and peroxiredoxin like 2A (PRXL2A) [Figure 8].

### EV-mediated modulation of LPS-stressed ECs (LPS/EV *vs.* LPS)

To assess the specific effect of EV treatment on LPS-stressed cells, we focused on the LPS/EV *vs*. LPS comparison, which isolates the proteome changes attributable to EV incorporation independent of the underlying LPS stimulus. This comparison identified 121 differentially expressed proteins (*P* < 0.05, |log2 fold change| ≥ 1), of which 63 were upregulated and 58 downregulated by EV treatment [Figure 4B]. Among these, 44 proteins were exclusively significant in this comparison (not differentially expressed in either LPS *vs*. ECs or LPS/EV *vs*. ECs), representing a focused set of EV-specific candidates [Figure 2A and Supplementary Table 1].

**Figure 9:**
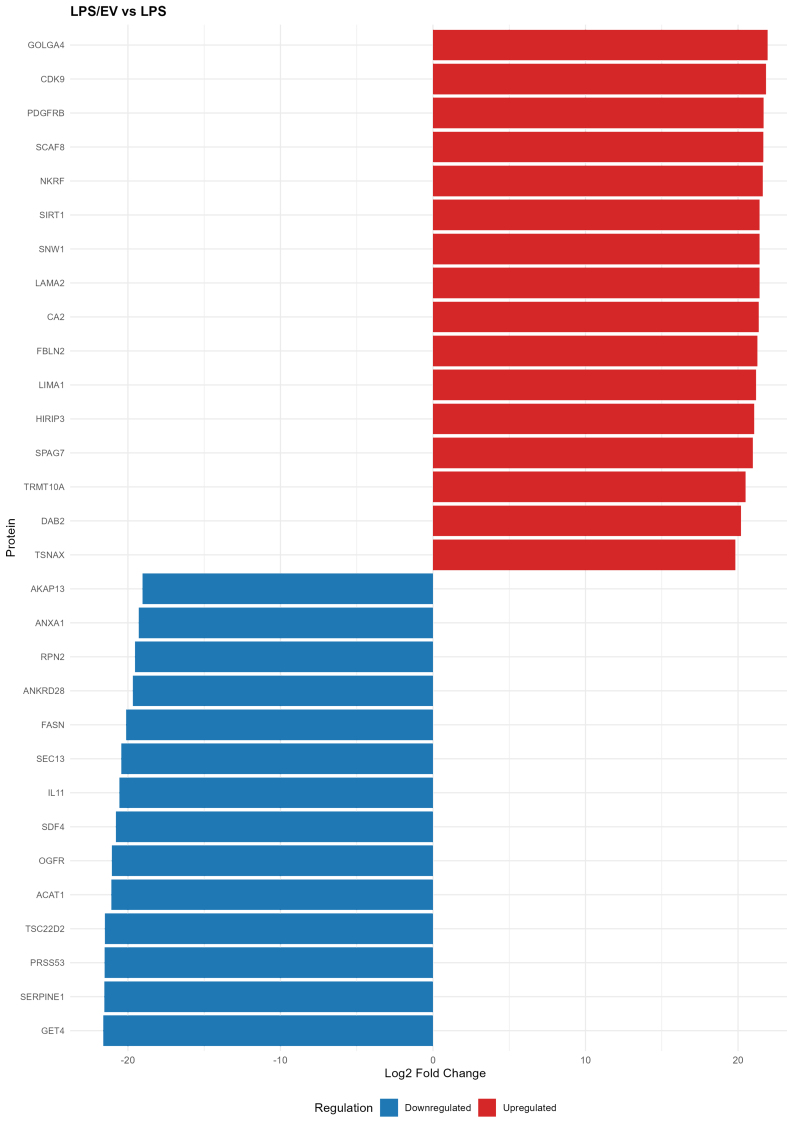
Bar chart showing the top 30 differentially modulated proteins by EV treatment, from the LPS/EV *vs*. LPS comparison. Red bars indicate upregulated proteins, and blue bars indicate downregulated proteins. The x-axis shows log2 fold change values. EV: Extracellular vesicles; LPS: lipopolysaccharide; LPS/EV: lipopolysaccharide plus extracellular vesicles combined treatment group; log2FC: log2 fold change.

A chart of the 30 most significant proteins strictly regulated by EV treatment is shown in Figure 9, while a complete heatmap of all 121 significant LPS/EV *vs*. LPS changes is provided in Supplementary Figure 2.

### Functional analysis of EV-specific protein modulation

To characterize the biological significance of EV-mediated proteome changes, proteins from the LPS/EV *vs*. LPS comparison were classified into functional categories. To properly distinguish EV-specific effects from the underlying inflammatory response, proteins were assigned to three regulatory categories based on their behavior across all pairwise comparisons:

Category A - General inflammatory response: Proteins significantly altered in both LPS and LPS/EV groups compared to untreated ECs, reflecting the LPS-induced response that persists regardless of EV treatment. These are presented as validation of the inflammatory model.

Category B - EV-specific modulation: Proteins exclusively or differentially regulated in the LPS/EV *vs*. LPS comparison. Only these proteins are discussed as demonstrating EV-mediated effects.

Category C - EV-counteracted LPS effects: Proteins where the direction or magnitude of change in LPS/EV differs significantly from LPS alone (e.g., a protein upregulated by LPS but are restored toward baseline by EV treatment). These represent the most compelling evidence of EV anti-inflammatory action.

### Anti-inflammatory response

Among the 6 significant anti-inflammatory proteins identified, three of them (ARG2, NCOA5 and NR2C2AP) were upregulated in both LPS and LPS/EV groups compared to control ECs (Category A), representing a general cellular response to stress conditions that is not specifically triggered by EV treatment. These shared changes are consistent with a conserved endometrial stress response.

In contrast, EV treatment specifically modulated the expression of two proteins (Categories B and C). The expression of NUMA1, whose expression was reduced by LPS, was restored to control levels by EV treatment (Category C), suggesting a counteracting effect on LPS-induced stress. Conversely, Annexin A1 (ANXA1) was downregulated by EV treatment (Category B), suggesting that EVs may shift ECs toward a post-resolution phenotype in which the active anti-inflammatory signaling driven by ANXA1 is no longer required [Figure 7].

### Pro-inflammatory response

The pro-inflammatory response comprised 24 significantly deregulated proteins. Of these, the majority were differentially expressed in both LPS and LPS/EV groups compared to untreated ECs (Category A), reflecting the underlying inflammatory stimulus common to both conditions. Specifically, LPS upregulated several key mediators including GLYR1, AHCY, ASS1, IFIT3, IFI35 and IRF2BP2, while downregulating others such as Adenosine Deaminase RNA Specific B1 (ADARB1), X-Ray Repair Cross Complementing 1 (XRCC1), Solute Carrier Family 39 Member 7 (SLC39A7), IRF3, C1QBP and NKRF. These changes persisted in the LPS/EV group, indicating that 3 h of LPS stress followed by 24 h of EV treatment did not fully reverse the broad inflammatory proteome shift.

Crucially, EV treatment specifically modulated the expression of four proteins within this category (Categories B and C). The most notable was the downregulation of IL-11 by EV incorporation (Category B). Similarly, carnosine N-methyltransferase 1 (CARNMT1) was downregulated by EV treatment (Category B). Conversely, EV internalization led to the upregulation of a mannose-binding lectin [lectin mannose binding 2 (LMAN2), Category B] and, notably, of NKRF expression, initially reduced by LPS, was restored by EV treatment (Category C) [Figure 6].

### Oxidative stress resistance

The oxidative stress response comprised 41 significantly deregulated proteins. Again, the majority of LPS-induced alterations (TMX1, TXNDC9, MCM2, MCM3, MCM5, INTS3, PDCD11, ATP1B3, PRXL2A) persisted in the LPS/EV group relative to untreated ECs (Category A), reflecting the ongoing oxidative challenge from prior LPS exposure.

EV treatment specifically modulated the expression of several oxidative stress-related proteins (Categories B and C). EVs enhanced the expression of superoxide dismutase 2 (SOD2, Category B), activating signal cointegrator 1 complex subunit 1 (ASCC1, Category B) and platelet derived growth factor receptor beta (PDGFRB, Category B). Moreover, thioredoxin related TMX1, already overexpressed by LPS, was further enhanced by EV treatment (Category C), suggesting the reinforcement of adaptive mechanisms aimed at coping with oxidative stress and ER-associated protein misfolding.

Conversely, EV internalization reduced the expression of opioid growth factor receptor (OGFR), ATPase Na+/K+ Transporting Subunit Alpha 1 (ATP1A1), and integrin alpha-5 (ITGA5), whose expression was initially upregulated by LPS and brought back to control EC levels by EV internalization (Category C) [Figure 8].

### Additional EV-modulated proteins

The EV-specific effect also comprised the modulation of other proteins, including Sloan–Kettering Institute (SKI)-interacting protein [SKIP/SNW domain-containing protein 1 (SNW1)], Acetyl-CoA Acetyltransferase 1 (ACAT1), and extracellular matrix (ECM) proteins such as Laminin Subunit Alpha 2 (LAMA2) and fibulin 2 (FBLN2), as well as Serpin Family E Member (SERPINE1) [Figure 9].

## DISCUSSION

Endometrial inflammation can result in severe conditions that unbalance the uterine environment and competence, thus impairing the fertility of affected mares. The need for alternatives to canonical therapies has led to the way of the exploration of regenerative medicine strategies. In this context, EVs derived from extra fetal adnexa, such as the amniotic membrane, have emerged as promising candidates.

Equine ECs are able to incorporate amniotic EVs^[15]^. Amniotic EV incorporation has been examined with particular attention to the role of surface glycans on both EVs and target cells. Results demonstrated that surface glycans are involved in the internalization of AMC-EVs by eECs, with fucosylated and sialylated glycans playing a major role in this process.

Once internalized by ECs, amniotic EVs have been previously demonstrated to promote inflammation resolution in an *in vitro* model^[14]^. To mimic an inflammatory condition, ECs were exposed to 10 ng/mL LPS for 3 h. Amniotic EV internalization counteracted LPS-induced stress by reducing the expression of TNF-α, IL-6 and IL-1β when EVs were added either before, simultaneously with or after LPS challenge^[18,19]^. The miRNA cargo of these EVs revealed the presence of miRNAs involved in immunomodulation and inflammatory response^[16]^. More recently, amniotic EVs have also been successfully applied to the treatment of equine reproductive pathologies such as endometritis^[17]^.

Considering the encouraging findings and the effectiveness of these models, further investigation was undertaken to deepen the understanding of the anti-inflammatory effects of amniotic EVs on LPS-stressed ECs. Beyond miRNA-mediated mechanisms, the contribution of EV-associated proteins and their potential transfer to ECs during inflammatory processes were explored using a proteomic approach. Following the methodology described by Perrini *et al*.^[14]^, equine ECs were first stressed with 10 ng/mL LPS for 3 h and subsequently incubated with 400 × 10^6^ EVs/mL for 24 h (according to Gaspari *et al*., 2025^[15]^).

EVs were characterized following the MISEV guidelines^[10]^. Based on the dimensions detected by NTA analysis and TEM, the isolated nanoparticles can be classified as microvesicles. Moreover, they expressed canonical internal (Alix) and external markers (CD81 and CD63) and were negative for markers typical of cell lysates (Calnexin). In addition, TEM analysis further confirmed a well-defined vesicular morphology with no evidence of protein precipitates.

Our results identified a total of 3,307 proteins, of which 794 were differentially expressed across all experimental conditions. From PCA, a clear separation is evident between ECs and the other two groups, which are also distinct from each other, albeit along the component explaining a lower proportion of variance. This finding is not unexpected, as EV internalization - despite being intended to counteract LPS effects - nonetheless represents a perturbation of the physiological cellular state.

GO enrichment analysis was performed to investigate the response elicited in ECs by LPS stress and subsequent EV treatment. As expected, the comparison between LPS and ECs groups revealed enrichment of terms related to stress response, oxidative regulation, and signaling of TNF and NF-κB factors. Although seemingly predictable, these data provide strong support for the validity and robustness of this endometrial inflammatory model. The set of GO terms enriched in the comparison between LPS/EV and ECs groups closely overlaps with those from LPS group, which is expected and consistent with the fact that these cells were subjected to LPS exposure prior to EV treatment. However, this comparison also revealed enrichment of vesicle-related terms, once more confirming the actual internalization of EVs into ECs. Notably, vesicle uptake by equine ECs was not directly assessed in this study but has been extensively demonstrated in previous works^[14,15]^.

Only the LPS/EV versus LPS comparison reflects the direct “EV effect” on LPS-induced inflammation. Vesicle internalization led to the upregulation of proteins associated with more GO terms related to the anti-inflammatory response, including wound healing and negative regulation of apoptosis processes.

Thereafter, the analysis was focused on differentially regulated proteins involved in pro- and anti-inflammatory responses and in oxidative stress resistance. As expected, compared to control ECs, LPS challenge elicited marked expression alterations in proteins involved in the inflammatory and oxidative response.

Among pro-inflammatory proteins, LPS treatment increased the expression of GLYR1 (also knowns as NP60), which regulates the stress-induced activation of p38alpha, a broadly expressed signaling molecule with essential roles in inflammatory cytokine induction and apoptosis^[28]^.

Another upregulated protein was AHCY, which regulates intracellular S-adenosylhomocysteine (SAH) concentration can influence pro-inflammatory signaling through DNA methylation; elevated AHCY levels have been positively correlated with chronic inflammatory conditions^[29]^. Additionally, ASS1, reported as a pro-inflammatory gene^[30]^ was also upregulated. The LPS-induced upregulation of ASS1 indicates metabolic remodeling consistent with pro-inflammatory macrophage activation, as citrulline depletion by ASS1 is required for sustained inflammatory responses^[30]^. This enzyme catalyzes the penultimate step of the arginine biosynthetic pathway. Given its link with the arginine-nitric oxide (NO) pathway, ASS1 upregulation indirectly suggests an increased NO release^[31]^, a typical pro-inflammatory mediator. Elevated NO levels have been detected in the uteri of mares susceptible to endometritis^[32,33]^. Interestingly, despite expectations, a direct increase in inducible nitric oxide synthase expression was not detected in the present study.

LPS stress also altered the expression of interferon-induced and related proteins such as IRF2BP2, IFIT3, IFI35 and IRF3, signaling pathway regulators involved in innate immune system response. Stressed ECs also displayed reduced expression of proteins associated with DNA repair mechanism (XRCC1), zinc transport and endoplasmic reticulum (ER) stress (SLC39A7)^[34]^, C1QBP, and transcriptional repression of NF-κB (NKRF).

Oxidative stress is a central driver of the morphological and functional alterations affecting endometrial tissue during endometritis^[35]^. Concerning oxidative resistance proteins, LPS induced an overabundance in thioredoxin family members and thioredoxin domain-containing proteins (TXNDC9, TMX1), which participate in redox regulation and may enhance NF-κB DNA-binding and transcriptional activity^[36]^. Additional increases were observed in DNA damage response proteins, such as minichromosome maintenance complex components (MCM2, MCM3, MCM5, MCM7)^[37]^ and INTS3, as well as NF-κB-binding proteins (PDCD11) and peroxiredoxins (PRXL2A), involved in the modulation of reactive oxygen species (ROS) synthesis via NADPH oxidase activity^[38]^. Moreover, the overexpression of Na/K-ATPase subunits (ATP1B3), which is responsible for establishing and maintaining the electrochemical gradients of Na+ and K+ ions across the plasma membrane, suggests disrupted ion homeostasis and oxidative stress-related signaling^[39]^.

Collectively, these changes reflect an active compensatory cellular response to LPS exposure and suggest the inflammatory response was sufficiently triggered by LPS stimulus. However, no increase in IL-6 or IL-1β was observed, in contrast to what was previously described^[14]^.

Conversely, the incorporation of EVs by LPS-stressed ECs revealed a counteractive effect on nuclear protein NUMA1 expression, whose levels were increased by EV treatment. The NUMA1 protein is a key structural component central to nuclear formation, mitotic spindle assembly and positioning and general nuclear matrix integrity^[40]^. Its restoration by EV treatment may reflect the recovery of normal cellular architecture disrupted by LPS. Recent data suggest NUMA1 also participates in transcriptional response and repair of oxidative DNA breaks at non-coding regulatory regions^[41]^. Given the core role of this protein during mitosis, its proper function is essential for proper cell division. Speculatively, an enhancement in NUMA1 expression by EV incorporation might reflect a pro-proliferative effect of EVs on ECs, potentially corroborating previous findings^[14]^.

Conversely, EV internalization led to the down-regulation of ANXA1. Annexins are a family of calcium-dependent phospholipid-binding proteins, which are known for their role in regulation of inflammation, cell proliferation, differentiation, apoptosis, membrane trafficking and organization^[42,43]^. They are also present at the fetal-maternal interface and involved in the maintenance of optimal microenvironment for implantation^[44]^. Nevertheless, despite ANXA1 well-established anti-inflammatory role, controversial findings regard its pro-inflammatory activity^[45]^. Emerging evidence revealed its involvement in inflammatory-mediated diseases and cancer development, where ANXA1 expression levels show correlation with disease severity and cancer invasiveness and proliferation^[46]^. Moreover, increased levels of ANXA1 were detected in human endometriotic lesions, suggesting its involvement in the pathogenesis of endometriosis^[47]^. Conversely, in a murine model of acute LPS-induced endometritis, a complete lack of ANXA1 was found to deregulate local, systemic and inflammatory responses, exacerbating the production of cytokines and cellular recruitment to the endometrium^[48]^. Therefore, the down-regulation of this protein following EV treatment still presents challenges for interpretation. Given its well-established role as a pivotal mediator of the resolution phase of inflammation, EVs may shift ECs toward a post-resolution phenotype in which the active anti-inflammatory signaling driven by ANXA1 is no longer required^[47]^.

Moreover, EV internalization resulted in decreased levels of IL-11. Although IL-11 exerts immunomodulatory effects by inhibiting TNF-α, IL-1β and IL-12^[49]^, it also activates Janus kinase/Signal Transducer and Activator of Transcription 3 (JAK/STAT3) signaling, increasing the expression of pro-inflammatory genes, and has been recognized as an inflammaging factor and member of the senescence associated secretory phenotype^[50]^. IL-11 overexpression in the endometrium has been associated with fibrosis and impaired tissue regeneration. Its downregulation upon EV internalization suggests a specific EV-mediated suppression of pro-fibrotic inflammatory signaling.

Similarly, CARNMT1 was down-regulated by EV treatment. This methyltransferase converts carnosine into anserine, which acting as metal ion-chelating agent serves as antioxidant and anti-inflammatory mediator; but CARNMT1 can methylate other target peptides as well^[51]^. Shimazu *et al*. showed that the presence of CARNMT1 led to an increase in the half-life of TNF-α mRNA and that CARNMT knock-out into macrophage cell line stimulated with LPS resulted in a reduction in TNF-α levels, suggesting the role of this methyltransferase into the enhancement of TNF-α expression during inflammation^[52]^. Therefore, its downregulation by EVs may contribute to reducing the inflammatory burden.

Conversely, EV treatment after LPS stress also led to the upregulation of LMAN2, a transmembrane lectin that binds high mannose glycoproteins. Consistently with the detection of high-mannose N-linked glycans on the surface of equine amniotic EVs^[15]^, the overabundance of LMAN2 might further promote the interaction of ECs with EVs. Considering that vesicle uptake is a dynamic rather than a fixed process, it might be speculated that an increase in these transmembrane lectins may reflect an active cellular response to the glycoprotein cargo delivered by EVs, aimed at further increasing EV internalization, facilitating their endosomal processing and intracellular trafficking.

Notably, vesicle uptake successfully counteracted the reduction in NKRF expression induced by LPS. NF-κB-repressing factor is a transcriptional repressor that binds to specific negative regulatory elements in the promoters of NF-κB target genes. This binding inhibits the transcriptional activity of NF-κB, thereby suppressing the expression of pro-inflammatory cytokines and enzymes. The activation of NF-κB is widely acknowledged to play a crucial role in the onset and progression of endometrial diseases^[53]^. Higher expression levels of NF-κB have been detected in the endometrium of chronic degenerative ednometritis (CDE)-affected mares^[54]^, promoting excessive deposition of ECM and contributing to implantation failure^[55]^. Activation of NF-κB signaling pathways resulted in the up-regulation of hyaluronan synthase activity, noted in destructive histopathological types of CDE^[54]^. Therefore, the expression of NF-κB inhibitors by ECs after AMC-EV internalization may contribute to taming the pro-inflammatory and pro-fibrotic endometrial environment typical of endometritis. Its restoration by EVs constitutes one of the most compelling demonstrations of EV-mediated anti-inflammatory action, as it suggests the re-establishment of a key negative feedback loop that restrains NF-κB-driven inflammation in the endometrium^[53,54]^.

Concerning the oxidative resistance-related proteins, EV uptake enabled to counteract LPS-induced effects by increasing the expression of ASCC1, which plays a critical role during DNA alkylation damage^[56]^ and SOD2, pivotal enzyme in ROS clearance and in the balancing of oxidative resistance.

Moreover, vesicle internalization also led to a further overabundance of TMX1, ER protein with a role in redox balancing and coping with protein misfolding under conditions of oxidative challenge and ER stress. The additional upregulation of this protein, beyond the overexpression already induced by LPS stimulation, may suggest reinforcement of adaptive resistance to oxidative stress and ER-associated protein misfolding.

Conversely, EV uptake reduced the expression of OGFR, regulator of cell proliferation and tissue organization, also involved in fatty acid oxidation^[57]^. Additionally, ATP1A1 was also down-regulated. As a member of the Na/K-ATPase, it is responsible for the exacerbation of oxidative stress due to the activation of membrane-bound Src kinase, which eventually leads to the activation of the downstream pathway of the Rat sarcoma (RAS)–Rapidly accelerated fibrosarcoma (RAF)–MAPK/ERK kinase (MEK)–Extracellular signal-regulated kinase (ERK) signaling pathway, resulting in excessive mitochondrial oxidation^[39,58,59]^.

Moreover, ITGA5 expression, initially upregulated by LPS, was also restored to control ECs levels by EV internalization. Main receptor of fibronectin, ITGA5 promotes the deposition of collagen fibers in the ECM; if excessively upregulated, this process leads to tissue fibrosis^[60]^, as observed in chronic endometritis.

Finally, the EV specific effect comprises also comprises the modulation of other proteins, including other transcription factors and co-regulators, mitochondrial enzymes (ACAT1) and ECM proteins, such as laminin (LAMA2) and fibulin (FBLN2) or metalloproteinase inhibitors (SERPINE1). The modulation of different ECM protein expression by EV internalization highlights their regenerative role, which is not limited to reducing inflammation, but is extended also to matrix remodeling and tissue organization. Indeed, an excessive or dysregulated ECM deposition reflects a pro-fibrotic state of the tissue, which compromises its functionality - as observed in chronic endometritis. Therefore, maintaining a balance between ECM deposition and its remodeling by matrix metalloproteinases is essential for the preservation of tissue homeostasis or its regeneration.

Vesicle internalization also increased SNW1 expression, a transcriptional co-regulator involved in mRNA splicing and TGF-/SMAD signaling^[61]^. SNW1 has a role also in the transcriptional elongation of some NF-κB target genes^[62]^ and has been identified as a possible marker of equine melanocytic neoplasm^[63]^, suggesting its pro-inflammatory action.

Additionally, PDGFRB was also upregulated by EVs. The response to PDGF can be modulated not only by the bioavailability of the ligand, but also by the expression of its receptor on cell surface^[64]^. Indeed, during inflammatory insults, PDGFRB can be upregulated in order to promote the action of its ligand PDGF. By regulating angiogenesis, inflammatory response, chemotaxis and matrix molecules deposition, PDGF finally promotes tissue repair^[65,66]^. Additionally, PDGFRB inhibition hampered uterine regenerative effect of MSCs in a murine model^[67]^, identifying this factor as an essential driver of endometrial tissue repair.

Altogether, these data offer an insight into the factors modulated within inflamed ECs following EV internalization. However, they represent a snapshot of EV-mediated effects 24 h post uptake - a timeframe sufficient for internalization, but likely insufficient to capture the full temporal dynamics of EV-driven intracellular responses. It is therefore plausible that the proteomic profile of ECs may evolve over time, reflecting a dynamic modulation of the anti-inflammatory and regenerative responses.

Furthermore, the same rationale may explain the overabundance of some classically considered pro-inflammatory proteins following EV treatment. Consistent with observations from other regenerative medicine products, EV treatment may transiently upregulate certain pro-inflammatory factors as an early recruitment phases of additional immune mediators.

A further limitation of this study lies in amniotic EV production itself. Despite extremely standardized, the protocol relies on the use of amniotic membranes, which are inherently variable even when collected from the same biological donor. This adds to the heterogeneity of the subpopulations composing MSC pool. As a result, the EV product obtained from each isolation may introduce a degree of variability, which could potentially influence downstream cellular responses.

Concluding, the anti-inflammatory effects of amniotic EVs on LPS-stressed ECs involve coordinated mechanisms, including cytokine signalling, antioxidant protection, transcriptional regulation, and ECM remodelling.

Therefore, this study provides insight into potential protein mediators underlying the anti-inflammatory response elicited by amniotic EVs in equine ECs during the early phase of exposure (24 h). Undoubtedly, further functional studies are required to validate the specific roles of the proteins identified in mediating EV therapeutic effects.

## DECLARATIONS

### Availability of data and materials

The dataset supporting the conclusions of this article are deposited to https://massive.ucsd.edu/ProteoSAFe/static/massive.jsp.

### Copyright

© The Author(s) 2026.
