# Supplementary material for: Amniotic extracellular vesicles’ effect on equine endometrial cells challenged with LPS: a proteomic analysis
Source: Extracell Vesicles Circ Nucl Acids. 2026 May 19;7(2):650–74. doi: 10.20517/evcna.2025.172 (PMC13367154; doi:10.20517/evcna.2025.172)
Supplement: Supplementary file 1 [file evcna-7-2-650-SupplementaryMaterials.zip › evcna60172-SupplementaryMaterials/evcna60172-SupplementaryMaterials.pdf]

Supplementary Materials

Amniotic extracellular vesicles' effect on equine endometrial cells challenged with LPS: a proteomic analysis

Giulia Gaspari[1,#], Alessio Soggiu[2,#], Paola Gagni[3], Salvatore Desantis[4], Fausto Cremonesi[1], Anna Lange-Consiglio[1]

[1]Department of Veterinary Medicine and Animal Science (DIVAS), Laboratory of Reproduction and Regenerative Medicine, Università degli Studi di Milano, Lodi 26900, Italy.

[2]Dipartimento di Scienze Biomediche, Chirurgiche e Odontoiatriche, Università degli Studi di Milano, Milan 20133, Italy.

[3]Istituto di Scienze e Tecnologie Chimiche "Giulio Natta" (SCITEC), Consiglio Nazionale delle Ricerche (CNR), Milan 20133, Italy.

[4]Department of Regenerative and Precision Medicine-Jonian Area (DiMePRe-J), University of Bari Aldo Moro, Bari 70124, Italy.

[#]These authors contributed equally to this work.

ORCID: Anna Lange-Consiglio (0000-0002-7748-3413)

Correspondence to: Prof. Anna Lange-Consiglio, Department of Veterinary Medicine and Animal Science (DIVAS), Laboratory of Reproduction and Regenerative Medicine, Università degli Studi di Milano, Lodi 26900, Italy. E-mail: anna.langeconsiglio@unimi.it

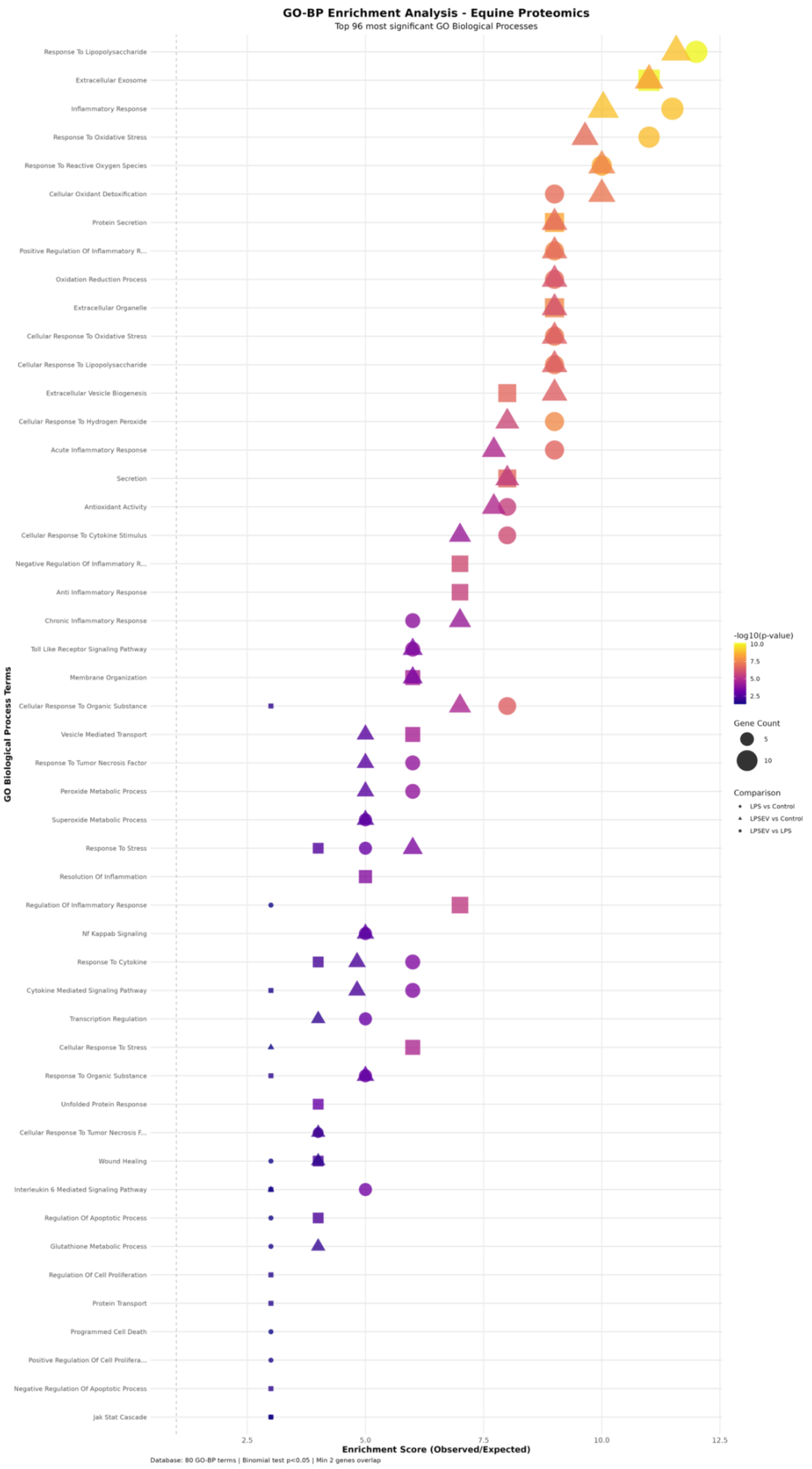

Figure 1. Most significant Gene Ontology Biological processes enriched among comparisons between LPS and control EC groups (circular dots), LPS/EV and control EC groups (triangular dots), LPS/EV and LPS groups (square dots).

Equine PURE EV EFFECTS Analysis
(121 proteins with significant LPSEV vs LPS changes)

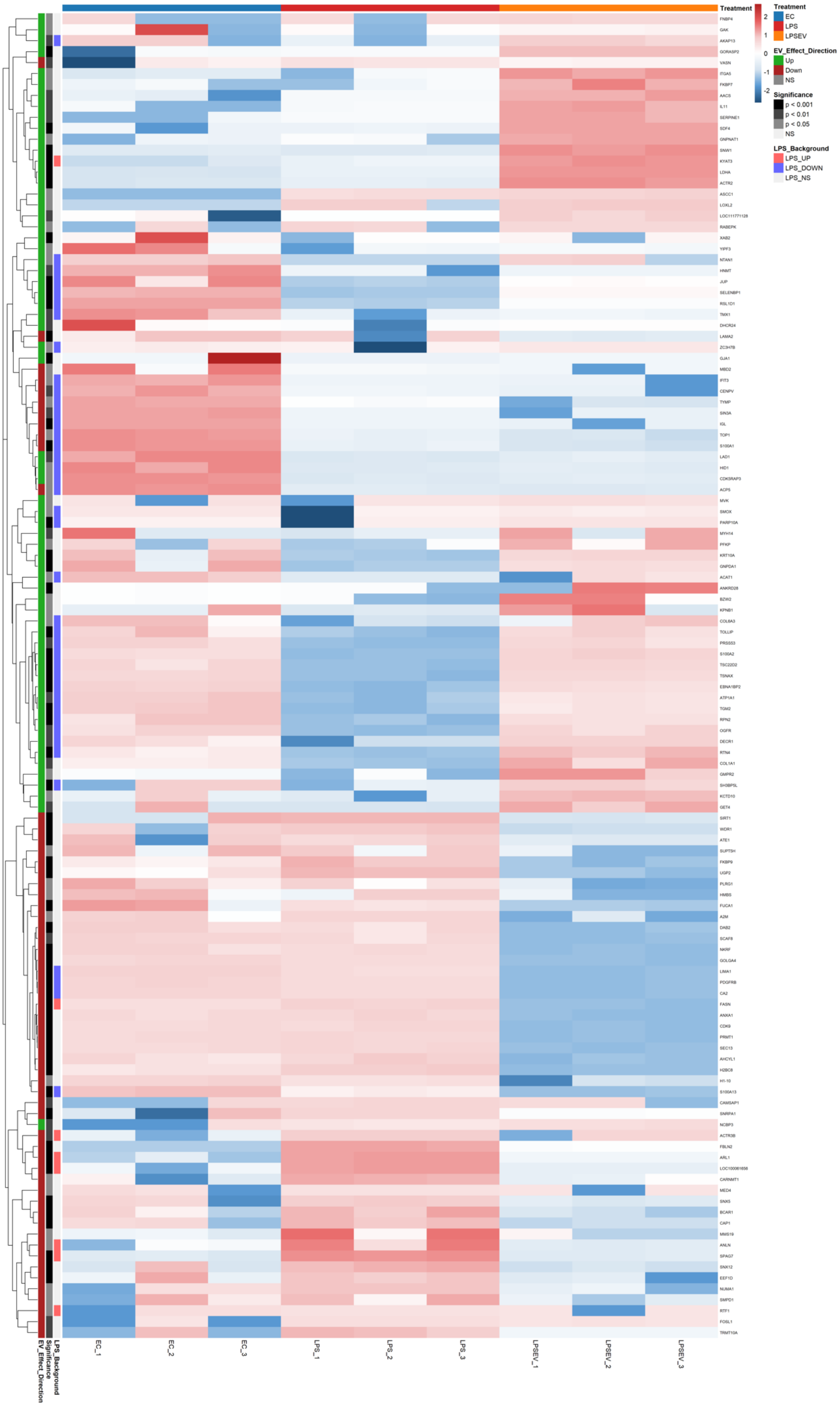

Figure 2. Heatmap representation of all 121 proteins with significant LPS/EV *vs*. LPS changes, representing the "pure EV effect". Z-score normalized expression values are displayed, with red indicating higher and blue lower relative expression. Left annotation columns: EV_Effect (green = enhanced by EVs, red = suppressed by EVs, white = not significant in LPS/EV *vs*. LPS comparison); Significance (in gray scale ranging from black = $P < 0.001$ to white = not significant); LPS_Response (red = upregulated by LPS, blue = downregulated by LPS, white = not significant in LPS *vs*. ECs comparison). Upper colored bars indicate experimental groups: light blue (ECs), red (LPS), orange (LPS/EV).
